# Design and Optimization of Heat Treatment Process Parameters for High-Molybdenum-Vanadium High-Speed Steel for Rolls

**DOI:** 10.3390/ma16227103

**Published:** 2023-11-09

**Authors:** Jibing Chen, Yanfeng Liu, Yujie Wang, Rong Xu, Qianyu Shi, Junsheng Chen, Yiping Wu

**Affiliations:** 1 School of Mechanical Engineering, Wuhan Polytechnic University, Wuhan 430023, China15965826765@163.com (Q.S.); chenjs9610@163.com (J.C.); 2 School of Materials Science and Technology, Yanshan University, Qinhuangdao 066004, China; 3 Powder Metallurgy Research Institute, Central South University, Changsha 410083, China; 4 School of Materials Science and Technology, Huazhong University Science and Technology, Wuhan 430074, China; yipingwu@hust.edu.cn

**Keywords:** high-molybdenum-vanadium high-speed steel, JMatPro, austenitic carbon content, martensite transformation temperature, heat treatment process

## Abstract

High-molybdenum-vanadium high-speed steel is a new type of high-hardenability tool steel with excellent wear resistance, castability, and high-temperature red hardness. This paper proposes a composition design of high-molybdenum-vanadium high-speed steel for rolls, and its specific chemical composition is as follows (wt.%): C2%, Mo7.0%, V7.0%, Si0.3%, Mn0.3%, Ni0.4%, Cr3.0%, and the rest of the iron. This design is characterized by the increase in molybdenum and vanadium in high-speed steel to replace traditional high-speed steel rolls with the tungsten element in order to reduce the heavy elements’ tungsten-specific gravity segregation caused by centrifugal casting so that the roll performance is uniform and the stability of use is improved. JMatPro (version 7.0) simulation software is used for the composition design of high-molybdenum-vanadium high-speed steel. The phase composition diagram is analyzed under different temperatures. The content of different phases of the organization in different temperatures is also studied. The martensitic transformation temperature and different tempering temperatures with the different types of compounds and grain sizes are calculated. The process parameters of heat treatment of high-molybdenum-vanadium high-speed steel are optimized. The selection of carbon content and the temperature of M50 are calculated and optimized, and the results show that the range of pouring temperatures, quenching temperatures, annealing temperatures, and tempering temperatures are 1360~1410 °C, 1190~1200 °C, 818~838 °C, and 550~600 °C, respectively. Scanning electron microscope (SEM) analysis of the samples obtained by using the above heat treatment parameters is consistent with the simulation results, which indicates that the simulation has important reference significance for guiding the actual production.

## 1. Introduction

Rolling mills perform the continuous plastic deformation of metal on working parts and tools. Various types of alloying elements have been explored and adjusted in rolled materials to meet the production requirements and advance rolling equipment toward large-scale, continuous, automated, high-speed development, as well as to improve the strength of rolled materials and increase their resistance to deformation. With advancements in industrial technology, the demand for steel has increased. Therefore, there is significance in research on improving rolled materials to meet production needs. Research on high-carbon and vanadium high-speed steel has shown [1] that high-speed steel not only has high strength and high hardness but also high thermal hardness and wear resistance. After quenching, its hardness can reach 63–70 rockwell hardness (HRC), retaining a high degree of hardness and abrasion resistance after tempering at 550–600 °C. For these reasons, high-speed steel is mainly used in manufacturing metal-cutting tools [2]. In addition to matrix hardness, high-carbon and -vanadium high-speed steel also has alloy carbides known as MC carbides in the reinforcing phase, producing vanadium-containing high-speed steel with high wear resistance (cast iron wear resistance and service life can be increased eight-fold compared to high chromium). It is widely used in producing wear-resistant parts of high-wear rolls, hammerheads, and other wear parts [3,4].

In addition to the development of rolls, China is paying more attention to production. In the 21st century, China’s development of rolls has focused not only on production but also on improving product quality to >95% [5,6]. However, increasing the production capacity of high-speed steel rolls used in more demanding environments and conditions is difficult because they are subject to key process parameters. Nippon Steel has high-speed steel with high vanadium content. The service life of these high-vanadium high-speed steel rolls is three times that of ordinary rolls [7,8]. Tang et al. [9] and BarZan D. [10] studied the abrasive wear properties of vanadium-containing high-speed steel by transmission electron microscopy and developed high-vanadium high-speed steel composite guide wheels, hammerheads, and grinding rolls. The electroslag remelting (ESR) and Continuous Pouring Process for Cladding (CPC) methods developed in Japan [11] and the Compact Cassette Rolling (CCR) method developed in the United Kingdom are present-day roll-manufacturing methods. In 1992, Canada’s Dofasco company began manufacturing 22 different types of high-speed steel rolls in 9 factories over 6 years. Many racks have been used in high-speed steel rolls. These high-speed steel rolls significantly increased the mill’s operating rate and improved the strip steel’s surface quality by 20% [12]. Based on Belgium’s Marichal Ketin company, South Africa’s Vanderbijlpark plant 2050 mm hot strip mill developed HSS7 high-speed steel rolls, with millimeters of steel rolled by high-chromium cast iron rolls weighing from 5000 tons to 12,000 tons. They also improved the surface quality of the hot-rolled strips [13].

High-molybdenum-vanadium high-speed steel belongs to a new type of high-C, high-Mo, high-V, and high-hardenability steel tool with good high-temperature red hardness, casting, molding, and abrasion resistance. However, there are few reports and studies on this steel, with no accurate description of its suitable working environment, chemical composition, or hot working process parameters (casting temperature, annealing temperature, and quenching temperature range). In this paper, the simulation can predict and determine the optimum hot working process routes and parameters, as well as provide the theoretical basis for formulating the manufacturing process for mill rolls and roll rings, which is particularly important for selecting the tempering temperature.

## 2. Research Content and Methodology

The subject of our research was high-molybdenum-vanadium high-speed steel. In our study, we investigated the mill roll rings’ working conditions. We selected or designed a suitable composition for high-molybdenum-vanadium high-speed steel mill roll ring materials and determined the most economical and reasonable hot processing route. Our research goals included the following:

(1) Determining the organizational composition of high-molybdenum–vanadium high-speed steel cold-rolled rolls and theoretically determining the alloy system by using empirical formulas and discussing the literature.
(2) We studied alloy system composition data with JMatPro (version 7.0) materials using simulation software for simulation calculations, including thermodynamic calculations, phase diagram analysis of the alloy composition, thermal property calculations, solidification calculations, hardenability calculations, high-temperature strength calculations, flow stress analysis, and fatigue analysis, to optimize and select the optimal set of material properties for a group of data.
(3) Optimizing heat treatment process parameters for roll rings fabricated from high-molybdenum-vanadium high-speed steel, selecting the optimal set of high-speed steel composition data for the phase transition calculation, drawing the Time Temperature Transformation/Continuous Cooling Transformation (TTT/CCT) curve, calculating the martensite transformation temperature, determining the quenching performance, determining energy changes, and developing the optimal heat treatment route.
(4) Drawing general conclusions on the virtual manufacturing of high-molybdenum-vanadium high-speed steel mill roll ring materials. We summarized the analysis and discussion to establish the optimum chemical composition and heat treatment process parameters.

We relied on the phase calculation platform in JMatPro (version 7.0) simulation software to analyze the solidification process of high-molybdenum-vanadium high-speed steel, the law of phase change, and the optimal casting temperature. We also analyzed the effects of annealing and quenching temperatures on the microstructure and performance to determine the optimal ranges of high-molybdenum–vanadium high-speed steel, casting temperature, and annealing and quenching temperatures. Providing a theoretical basis for selecting the hot processing parameters for these types of materials was also important.

## 3. High-Molybdenum-Vanadium High-Speed Steel Composition Selection and Phase Diagram Analysis

### 3.1. Selection of Compositional Data for High-Molybdenum–Vanadium High-Speed Steel for Rolling Mill Roll Rings

#### 3.1.1. Selection of Main Alloying Element Contents

The main alloying elements of high-speed steel for mill roll rings are tungsten, molybdenum, vanadium, and chromium, and the total amount of alloy can be as high as 15–20% [14].

In high-speed steel tungsten, molybdenum’s chemical properties are similar to carbide-forming elements and its role is nearly the same. It can improve red hardness, and its impact on the organization of high-speed steel transformation and performance is nearly the same. The main difference lies in the molybdenum created during transformation at lower temperatures [15]. Tungsten-containing high-speed steel in the heating and holding process has shown an increase in *W*_(W)_ content. It also improves dispersion strengthening and solid solution strengthening. Thus, tungsten can improve the thermal stability of high-speed steel [16]. The influence of tungsten on the microstructures and properties of high-speed steel is not proportional to its content. High-speed steel *W*_w_ at 7–8% can satisfy secondary hardness and thermal stability [17], but the carbide phase containing M_23_C_6_ carbides is excessive, and that containing M_6_C carbides is too little. A quenching temperature that is too high will produce very coarse grains and a significant decline in strength and toughness. M_6_C carbides increase with an increase in *W*_(W)_, which significantly improves the superheat stability of the steel. However, if *W*_(W)_ is too high, the roll microstructure in the leucite amount increases, causing carbide particles to be large and unevenly distributed, adversely affecting the thermal fatigue performance of the roll. The roles of molybdenum and tungsten are similar. Molybdenum also forms M_6_C carbides, with the same tungsten-containing high-speed steel in M_6_C carbides compared to its lattice. Furthermore, the lattice parameters are almost the same, that is, the density is lower than tungsten, which eliminates the phenomenon of segregation to some extent. Molybdenum in high-speed steel rolls can improve the inhomogeneity of primary carbides and reduce brittleness. Tempering molybdenum’s solid solution can prevent carbide precipitation along the grain boundaries and improve strength and toughness. Therefore, we aimed to replace the tungsten element in traditional high-speed steel rolls by increasing the content of several molybdenum elements in high-speed steel. We also sought to reduce tungsten in centrifugal casting caused by segregation to make the radial properties of the rolls uniform and improve the rolls’ stability.

Considering the comprehensive analysis above, our study is characterized by high-molybdenum high-speed steel with no added tungsten elements: tungsten content *W*_(W)_ = 0%, and molybdenum content *W*(_Mo_) = 7.0%. At this time, the tungsten equivalent of *W*_(Weq)_ =*W*_(W)_ + 2*W*_(Mo)_ = 14% < 15%, thus ensuring that the high-speed steel has good wear resistance and toughness.

Vanadium has a strong affinity for carbon; thus, increasing the amount of *W*_(V)_ in high-speed steel is equivalent to shifting all the points in the solidification phase diagram to the left. As the amount of *W*_(V)_ increases, the eutectic reaction temperature of high-speed steel decreases [18], and the formation temperature of MC carbides increases. Vanadium not only favors the formation of MC carbides but also promotes the formation of lamellar M_2_C carbides and inhibits skeletal M_6_C carbides. MC carbides have the highest hardness and high-temperature austenitization in various high-speed steel carbides. When MC carbide dissolution is difficult, it exists in the remaining phase, which is conducive to grain refinement and improves wear resistance. After the amount of *W*_(V)_ in high-speed steel rolls exceeds 8% [19], low-hardness M_3_C carbides appear in the eutectic organization, reducing the wear resistance of high-speed steel rolls. In addition to purifying steel, vanadium in high-speed steel rolls reduces inclusions and gas content and improves high-speed steel rolls’ resistance to thermal shock [20]. Therefore, the amount of *W*_(V)_ in high-speed steel rolls is kept between 3.0 and 8.0%. Thus, the vanadium content of high-speed steel designed in this study was *W*_(V)_ = 7.0%.

Chromium plays a significant role in preventing oxidation and preventing carbon from forming M_7_C_3_ carbides, which improves the wear, heat, and oxidation corrosion resistance of the matrix. Chromium promotes the precipitation of fine M_23_C_6_ carbides in the matrix [21], which can improve hardness. Lee et al. [22] studied the effects of different chromium levels on the organization and properties of high-speed steel, noting that, in similar components, the increase in chromium content and eutectic carbides in the matrix creates fine chromium-rich carbides. However, the impact on the primary carbide composition is minor. In addition, chromium is partially present in M_6_C carbides of high-speed steel, which can also form M_23_C_6_ carbides. M_23_C_6_ carbides can be completely dissolved at lower quenching temperatures, allowing the solid solution to reach carbon–chromium saturation without affecting grain size. Chromium can also promote austenite in completely dissolved M_6_C carbides, thereby improving the hardenability and red hardness of high-speed steel. In high-speed steel tempered at 450–525 °C, part of the chromium precipitation from the martensite promotes diffuse hardening. The other part of the chromium retained in the α-solid solution prevents softening when heated to higher temperatures. The addition of *W*_(Cr)_ 3–4% can obtain slightly higher secondary hardness. Chromium in high-speed steel can also reduce oxidation. When the *W*_(Cr)_ amount is too high, excess chromium is involved in tempering carbide precipitation. Chromium-containing carbides are easy to precipitate at lower temperatures, reducing the thermal stability of high-speed steel. It has been found [23] that chromium increases the number of M_7_C_3_ carbides and decreases the number of MC carbides in high-speed steel rolls, which is detrimental to wear resistance. Rolls with low *W*_(Cr)_ can develop rough surfaces due to preferential abrasion of the substrate and the rolled materials’ adherence to the roll surface, increasing the friction coefficient between the rolls and the rolling force. Roll surface roughness can be improved by increasing the amount of *W*_(Cr)_ and reducing the rolling force so that the roll contains a certain amount of M_7_C_3_ carbides. *W*_(Cr)_ is also conducive to improving the thermal shock resistance of the high-speed roll. Therefore, the appropriate *W*_(Cr)_ content in high-speed steel rolls is between 3.0 and 7.0%. Accordingly, the chromium content of high-speed steel designed in this study was *W*_(Cr)_ = 3.0%.

#### 3.1.2. Selection of Other Element Contents

We added small amounts of other elements to high-speed steel, such as silicon, manganese, nickel, etc., to improve its performance.

Silicon is a non-carbide-forming element. It can promote the formation of high-speed steel solidification in conventional high-speed steel by reducing harmful M_2_C in the re-heating decomposition of M_6_C and MC carbides, which eliminates columnar M_2_C carbides [24] to refine carbides and improve toughness. Silicon also increases the number of carbides in high-speed steel and improves the role of high-speed steel wear resistance. In addition, silicon can also refine the secondary carbides precipitated during tempering, improving tempering hardness. However, silicon is mainly dissolved in the matrix, increasing matrix brittleness. High-speed steel rolls in silicon are harmful elements. The content is too high, and the roll surface is prone to cracking and spalling. The amount of w(Si) in the roll is maintained at <1.0% to facilitate batching and production [25]. The silicon content of high-speed steel in this study was w(Si) = 0.3%.

Manganese (Mn) is an element that causes the phase transition point of A_1_ temperature to decrease in high-speed steel. An increase in *W*_(Mn)_ reduces the thermal stability and secondary hardness of high-speed steel. *W*_(Mn)_ amounts in ordinary high-speed steel are generally controlled at ≤0.4% [26]. An increase in *W*_(Mn)_ also increases high-speed steel rolls’ brittleness and quenching cracking risk. Mn also causes high-speed steel austenite grain growth. The *W*_(Mn)_ amount in high-speed steel rolls must be maintained at ≤1.0% to facilitate dosage and production. In our study, the manganese content of high-speed steel *W*_(Mn)_ was 0.3%.

Ordinary high-speed steel does not contain added nickel because it will increase the amount of residual austenite. However, the addition of nickel is also known to improve the mechanical properties of high-speed steel, especially its toughness and plasticity, by reducing the thermal conductivity of high-speed steel and the A_c1_ temperature. Kim et al. [27] found that adding nickel to high-carbon high-speed steel significantly decreased high-temperature strength, room-temperature strength, and hardness. However, an appropriate amount of added nickel improves the resistance of the rolls to cracking and spalling. Nevertheless, its effect on high-speed steel rolls’ strength is minor under the assumption that the matrix is martensitic. Nickel improves the annealed hardness of high-speed steel rolls [28], affecting the machinability of the rolls. Therefore, *W*_(Ni)_ content should be maintained at between 0.3 and 1.5%. The nickel content of high-speed steel in this study was *W*_(Ni)_ = 0.3%.

#### 3.1.3. Selection of Carbon Content

Carbon is the most important constituent element in high-speed steel, which significantly influences every aspect of high-speed steel manufacturing. Its mechanism of action involves the formation, transformation, dissolution, precipitation, and aggregation of carbides [29,30]. It can form MC, M_2_C, M_3_C, M_6_C, M_23_C_6_ carbides, and other carbide types with all of the strong carbon compounds of high-speed steel. It can also improve the performance of high-speed steel after forging, heat treatment, and other processes [31].

The general carbon content of high-speed steel rolls should be between 1.8 and 2.8%. Quenched and heated carbon parts are dissolved in austenite to ensure the hardness of martensite. Part of the alloy carbides undergo diffuse precipitation during tempering, resulting in secondary hardening, which prevents grain growth and increases steel hardness, abrasion resistance, and red hardness. The phase transition process cannot be finished if the carbon content is insufficient, and the finished product will not meet the expected results. However, too high of a carbon content will lead to high-speed steel quenching of residual austenites produced by increasing steel performance, reducing the need for multiple tempering treatments. Therefore, the carbon content of high-speed steel should be based on molybdenum, tungsten, vanadium, chromium, and other elements to determine the carbide ratio of the relationship between the steel to meet all the carbide-forming elements. In other words, this achieves the “carbon balance” that produces the maximum secondary hardening effect so that high-speed steel performance can achieve the optimum. As early as 1964, Steven et al. [32] summarized high-speed steel *W*_(C)_ amounts based on research proposing the concept of “equilibrium carbon” and determined the amount of high-speed steel *W*_(C)_ using an empirical formula. Although the formula has been verified in several conventional high-speed steel compositions, it is not universal. In recent years, Duan et al. [33] provided an empirical formula for determining the amount of *W*_(C)_ for a multivariate white cast iron similar to a high-carbon high-speed steel roll:

*W*_(C)_ = 0.060*W*_(Cr)_ + 0.063*W*_(Mo)_ + 0.033*W*_(W)_ + 0.235*W*_(V)_ + 0.13*W*_(Nb)_ (1)

Combined with the known elemental content, the carbon content was calculated to be 2.266%. However, relying on the formula alone is not enough. Simulations through JMatPro (version 7.0) software were determined based on the phase transition’s effect on the actual properties. The equilibrium carbon difference was set at 0.5, with 0.1 as the data interval, according to the known elemental content, using JMatPro’s simulation calculation test. Table 1 shows the simulation calculation data.

**Figure 1:**
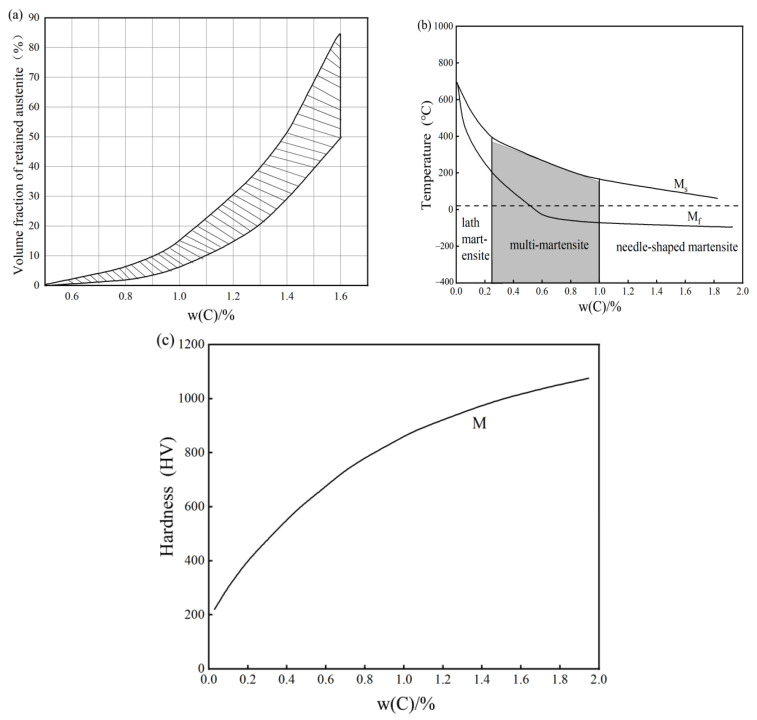
Relationship between carbon and some microstructures. (**a**) Effect of carbon mass fraction on retained austenite; (**b**) effect of carbon mass fraction on martensite morphology; (**c**) effect of carbon mass fraction on martensite hardness.

According to the literature [23,34,35], the amount of residual austenite and the temperature of martensite transformation are influenced by the mass fraction of carbon in the austenite. The martensite transformation start and termination lines are increasingly lower; however, with the increase in carbon content, the amount of residual austenite after tempering also increases, affecting the performance of high-speed steel. Increased carbon content in the martensite will lead to poor lamellar martensite performance and negatively impact the strength and hardness of high-speed steel strength based on the parabolic rule of change, as shown in Figure 1a–c. In summary, the carbon content in austenite should be maintained between 0.4 and 0.7%, and the higher the content in the appropriate range, the better.

In summary, according to Table 1, it can be seen that the optimal conditions are met when the carbon content is 2.0% and 2.1%. JMatPro (version 7.0) software was used to simulate and calculate the temperature values of the transformation of martensite to 50% (M50 (°C)) when the carbon content was 2.0% and 2.1%, respectively. The calculation results are shown in Table 2 and Table 3 below.

According to the analog data in Table 2 andTable 3, the austenite content increases with temperature before reaching its peak. The martensitic transformation temperature increases from 50% before gradually decreasing. Table 2 andTable 3 show that 2.0% and 2.1% carbon content in the material-quenching temperature reach 1230 °C and 1210 °C, respectively, when the M50 falls below 100 °C. Therefore, they must undergo a deep cold treatment to be made useful. The processing equipment requirements are high; thus, 50% of the martensitic transformation temperature should be controlled at 20–50 °C, while ensuring that the austenite content value is high. Comprehensive factors must be considered when comparing the two charts. For instance, what can be obtained with a carbon content of 2.0% is more reasonable. At the austenitization temperature of 1160 °C, the austenite content is 88.76%, the austenite carbon content is 0.69%, 50% of the martensitic transformation temperature is 150.7 °C, and the martensitic hardness is approximately 65 HRC.

Table 4 shows the composition content of high-molybdenum–vanadium high-speed steel according to the above discussion.

### 3.2. Phase Diagram Analysis of High-Molybdenum–Vanadium High-Speed Steel

#### 3.2.1. Analysis of Phase Composition Diagrams at Different Temperatures

**Figure 2:**
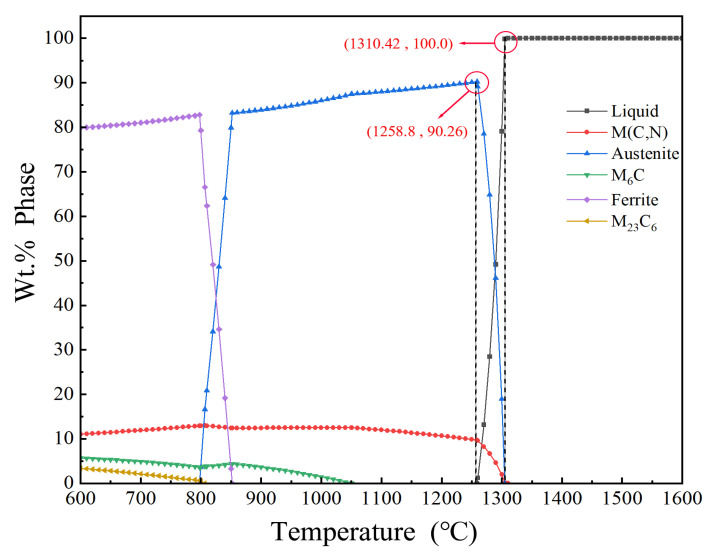
Equilibrium organization phase diagrams of Fe—3.0, Cr—0.3, Mn—7.0, Mo—0.4, Ni—0.3, Si—7.0, and V—2.0 C high-speed steel (numbers represent wt.%) at different temperatures.

Figure 2 shows the phase composition diagram of high-molybdenum-vanadium high-speed steel at temperatures ranging from 600 to 1600 °C using JMatPro’s temperature step calculation platform.

The alloy consists of three phase zones and three carbides, namely, the liquid phase zone, austenite, and ferrite phase zones, and M_6_C, M_23_C_6_, and M(C,N) carbides. The alloy is liquified at temperatures of >1310.42 °C, implying that the melting point of the alloy is 1310.42 °C, and the phase diagram shows the liquid-phase region. When the temperature is lowered, the M(C,N) carbides begin to precipitate slowly at 1305.04 °C because they mainly comprise VC carbides, which have high hardness and a high melting point. As the temperature decreases, the liquid-phase region of M(C,N) carbides gradually increases. When austenite appears, the alloy phase area from the liquid phase transforms into the austenite phase area. As the temperature decreases, austenite continues to increase alongside M(C,N) carbides. When the temperature is reduced to 1258.8 °C, the austenite content reaches a peak, implying that the liquid phase has completely transformed into the austenite phase. As the temperature decreases, the austenite content shows a slight downward trend, whereas M(C,N) carbides show a slightly higher trend. When the temperature drops to 1250.64 °C, the austenite phase begins to gradually precipitate into M_6_C carbides. As the temperature continues to fall, M_6_C carbides increase. When the temperature continues to drop to 852.06 °C, the austenite phase area begins to precipitate ferrite, which marks the beginning of the transition from the austenite to the ferrite phase. As the temperature continues to decrease, austenite gradually dissolves into ferrite, and the ferrite content rises sharply. When the temperature is reduced to 797.78 °C, austenite is completely transformed into the ferrite phase. At this time, the ferrite phase begins to gradually precipitate into M_23_C_6_ carbides. As the temperature drops again, four values tend to stabilize: The ferrite phase in the ferrite content, M(C,N) carbides content in the ferrite phase, M(C,N) carbides content, M_6_C carbides content, and M_23_C_6_ carbides content.

#### 3.2.2. Analysis of Solid and Liquid Phases

In Figure 2, high-molybdenum–vanadium high-speed steel is 100% in the liquid phase at >1310.42 °C; therefore, 1310.42 °C is the material’s melting point. M(C,N) carbides will slowly precipitate when the temperature drops below the melting point. When the temperature drops to 1305.04 °C, austenite will begin to precipitate as a result of the reaction between M(C,N) carbides and the liquid metal. As shown in Figure 2, when the austenite content reaches its maximum value at 1258.8 °C, the liquid phase completely disappears and the material austenitizes, creating the austenite solid-phase line.

### 3.3. High-Molybdenum-Vanadium High-Speed Steel Carbide Analysis

#### 3.3.1. Analysis of Elemental Content in Ferrite

**Figure 3:**
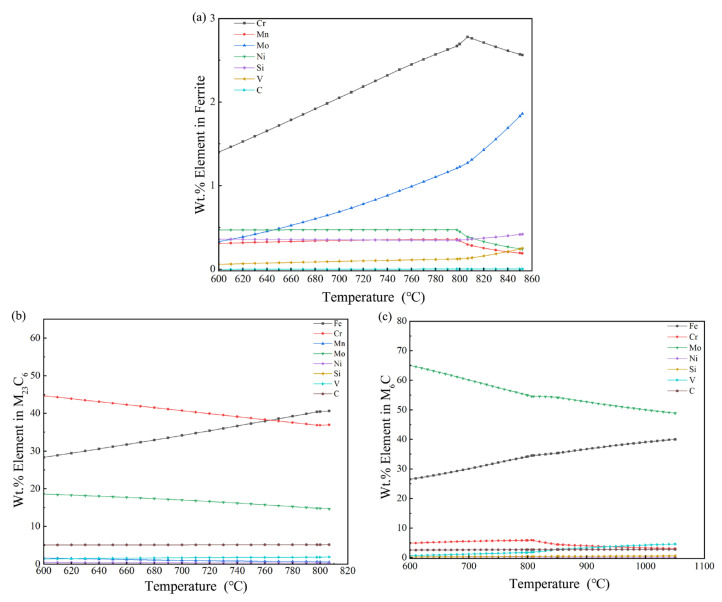
Content analysis of different carbides for Fe—3.0, Cr—0.3, Mn—7.0, Mo—0.4, Ni—0.3, Si—7.0, and V—2.0 high-speed steel. (**a**) Elemental content in ferrite; (**b**) elemental content in M_23_C_6_; (**c**) elemental content in M_6_C.

Ferrite is a phase region that alloy steel must pass through during the heating process. Figure 3c shows the change in content for each ferrite element according to JMatPro. When the temperature is between 600 and 810 °C, chromium content maintains its rising trend. When the chromium content reaches its peak at 806.55 °C, molybdenum content in the interval shows a straight line of upward growth. Manganese, silicon, and nickel levels in the ferrite phase are relatively stable and are conducive to the ferrite’s stabilization of these elements. Vanadium and carbon are very low trace elements in ferrite. Vanadium and carbon are also trace elements in ferrite with very low content and little change. Due to ferrite’s low concentration of dissolved carbon, carbon is mainly combined with other strong carbon compounds to produce M(C,N), M_6_C, and M_23_C_6_ carbides in this phase.

#### 3.3.2. Analysis of the Elemental Content of Carbon Compounds in M_23_C_6_ Carbides

Figure 3b shows a graph of the elements in M_23_C_6_-type carbides according to temperature change. This carbide type mainly consists of iron, chromium, and molybdenum elements. Chromium slowly decreases to between 600 and 800 °C, whereas iron increases. Molybdenum shows a slowly decreasing trend at approximately 18%. Carbon, vanadium, manganese, and nickel element contents are <5%, with no obvious change.

According to the high-molybdenum–vanadium high-speed steel phase diagram, M_23_C_6_ carbides mainly exist in the ferrite phase area at approximately 800 °C during the transformation of ferrite to austenite. Part of the M_23_C_6_ carbides and a small portion of ferrite occur in the encapsulation reaction generated by M_6_C compounds. The remaining M_23_C_6_ carbides in the quenching and heating process are dissolved in the austenite. M_23_C_6_ carbides in the matrix are granular, with a lower melting point of only approximately 840 °C. Its main role is to promote the secondary hardening effect in the tempering process.

#### 3.3.3. Analysis of M_6_C Carbides Carbon Compounds’ Elemental Content

Figure 3c shows that M_6_C carbides are mainly molybdenum carbides for elemental contents present in the temperature change curve. M_6_C carbides with respect to the molybdenum content have a decreasing trend during the ferrite phase’s transformation to the austenite phase at approximately 800 °C. Molybdenum content appears to have a short period of stabilization when the ferrite phase completely transforms to the austenite phase and continues to show a decreasing trend. The chromium content in M_6_C carbides is relatively low. The chromium content increases slowly from 4.86% to a peak of 5.89% when the temperature increases from 600 to 806.55 °C, and then decreases to 3.04% when the temperature increases to 1050 °C. The chromium content remains at 3% during the transformation process. The carbon content remains at the 3% level, combining with molybdenum and chromium to form M_6_C carbides. The vanadium content shows a rising trend throughout the process, and the growth rate reaches its maximum at the node of transformation from the ferrite to the austenite phase. The vanadium content in M_6_C carbides continues to increase with temperature and finally stabilizes at approximately 4.58%. The nickel and silicon content in M_6_C carbides is insignificant for this carbide type, remaining almost unchanged from 600 to 1050 °C. M_6_C carbides are also characterized by a high degree of austenitization, and the vanadium content in M_6_C carbides remains almost unchanged.

#### 3.3.4. Analysis of the Elemental Content of M(C,N) Carbides

**Figure 4:**
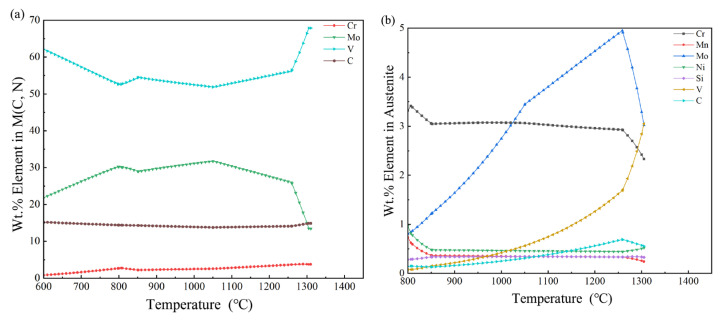
Content analysis of different carbides in Fe—3.0, Cr—0.3, Mn—7.0, Mo—0.4, Ni—0.3 Si—7.0, and V—2.0 high-speed steel. (**a**) Elemental content of M(C,N) carbides; (**b**) elemental content of austenite.

Figure 4a shows a temperature dependence graph for M(C,N) carbides’ elemental content from 600 to 1400 °C. The average vanadium content is >55%, indicating that this carbide type is dominated by vanadium carbides. Vanadium content shows a slow decreasing trend between the ferrite and austenite phase regions but increases slightly when the ferrite to austenite transformation occurs at approximately 800 °C. Then, the vanadium content reaches a peak value of 67.89% during the austenite-to-liquid phase transformation at approximately 1260 °C. The percentage of niobium rises considerably as most of the MC carbide elements transform into the liquid phase due to vanadium carbides’ high strength, hardness, and melting point properties. Out of all carbides in high-molybdenum–vanadium high-speed steel, vanadium carbide has the highest stability from 600 to 1260 °C. The impact of temperature changes on M(C,N) carbide content is approximately 1%, playing a major role in vanadium carbides. VC carbide cells have a face-centered cubic structure and small particles and are uniformly distributed on the austenite substrate. They cannot dissolve even under high-temperature quenching conditions, thereby improving high-speed steel’s hardness, strength, and wear resistance. Therefore, it is important to select the appropriate austenite content and ensure high vanadium carbide content when determining quenching temperatures.

Molybdenum is also present in large quantities of M(C,N) carbides. The molybdenum content increases slowly in the ferrite phase, decreases slowly as the ferrite dissolves into austenite, and then decreases sharply when the austenite phase changes into a liquid phase.

In addition to molybdenum and vanadium, a small amount of chromium is also present in MC carbides. Figure 4a shows that the chromium content remains almost unchanged throughout the whole process, indicating that the chromium and carbon formed by the M(C,N) carbides are more stable.

#### 3.3.5. Analysis of Elemental Content in Austenite

Figure 4b shows a graph of elemental contents in austenite versus temperature. Chromium and molybdenum are pro-carbide-forming elements that reduce the diffusion rate of carbon in austenite. The austenite phase in high-speed steel is mainly dominated by chromides from 800 to 1300 °C. Most of the chromides in the austenite begin to decrease at the beginning of austenite dissolution around 1250 °C. Molybdenum content shows a steady growth rate at 800 °C until peaking at 1250 °C, when it sharply declines. Vanadium content’s rate of increase is relatively low at low temperatures. From 1250 °C onward, its growth rate becomes faster until peaking at 1300 °C. Manganese and silicon content remain at ≤0.5%. Carbon is the main element affecting austenite performance. The carbon content slowly increases during austenite dissolution at 1250 °C and reduces during the liquid phase after the beginning of the transition.

High-molybdenum-vanadium high-speed steel belongs to over-eutectic steel, which is characterized by high carbon and alloys. This process forms carbides with alloying elements through a sufficient amount of carbon dissolved into austenite to obtain hardness and wear-resistant martensite after quenching. The austenite content directly determines martensite content after heat treatment. Therefore, quenching temperatures should be chosen based on high austenite content. As shown in Figure 2, almost all chromium content dissolved in the austenite during the final heating. Experiments have proven that the carbon content is higher in medium steel. Furthermore, chromium promotes austenite refinement and improves its strength and wear resistance.

## 4. Treatment Process Parameters and Analysis of High-Molybdenum-Vanadium High-Speed Steel Heat

### 4.1. Analysis of Solidification Process

As shown in Figure 2, high-molybdenum-vanadium high-speed steel at 1310.42 °C is completely transformed into the liquid phase when heated. The high melting point of vanadium carbides, a class of compounds, promotes hardness. Therefore, M(C,N)-type carbides from the liquid phase are the first to precipitate in the form of slowly precipitating particles covered by the matrix. Austenite starts to precipitate when the temperature is lowered to 1305.04 °C, reaching the solid phase point when the temperature is lowered to 1258.8 °C. Then, the liquid phase disappears completely, at which point the material consists of austenite and M(C,N) and M_6_C carbides.

To avoid solidification in the casting process, the casting temperature of the material is generally set above the liquid phase line at 50 °C to 100 °C. Therefore, the casting temperature of the material ranges from 1360 to 1410 °C.

### 4.2. Selection of Heat Treatment Process Parameters

#### 4.2.1. Quenching Temperature

Quenching is when the steel is heated to A_c1_ or A_c3_ above a certain temperature for a certain period of time to austenitize the material. This process is performed after the cooling rate exceeds the critical cooling rate for martensite transformation, improving the steel hardness of the heat treatment method. According to Figure 2 and the previous analysis, high-molybdenum–vanadium high-speed steel at A_c1_ = 797.78 °C and A_c3_ = 852.06 °C belongs to eutectic steel. Therefore, the quenching temperature should be greater than the A_c3_ temperature value.

Austenite carbon content is also an important criterion for determining quenching temperatures. As shown in Figure 1a–c, the austenite carbon content in austenite directly determines the carbon content of martensite, as well as the residual austenite content, martensite transformation temperature, and martensite hardness. As previously mentioned, carbon content between 0.4 and 0.7% gives the best performance. Therefore, the quenching temperature ranged from 1160 to 1300 °C.

The CCT curve was calculated using JMatPro for TTT/CCT diagrams ranging from 1160 to 1260 °C. The final quenching temperature was determined by the temperature at which 90% of the martensitic transformation occurred. The last column of Table 2 records the M90 temperature at different quenching temperatures. The quenching temperature increases as the martensitic transformation temperature of 90% decreases. The quenching temperature is higher than 1230 °C, whereas M90 is <0 °C, suggesting that the steel must have a deep-cooling treatment. High-molybdenum–vanadium high-speed steel production conditions can be achieved at room temperature. When the quenching temperatures were 1190 °C and 1200 °C, the temperatures of M90 were 30.4 °C and 21.0 °C, which meet production requirements.

#### 4.2.2. Annealing Temperature

Annealing eliminates the material’s residual internal stresses, prevents material deformation, improves cutting performance, and adjusts the hardness of the material. The process involves heating the steel to a certain temperature, maintaining it for a certain period of time, and then cooling it slowly with the heating furnace. Since high-molybdenum–vanadium high-speed steel belongs to eutectic steel, a spheroidal annealing process must be used to heat the steel to A_c1_ above 20–40 °C. The annealing temperature will be between 817.78 and 837.78 °C since A_c1_ = 797.78 °C.

#### 4.2.3. Tempering Temperature

**Figure 5:**
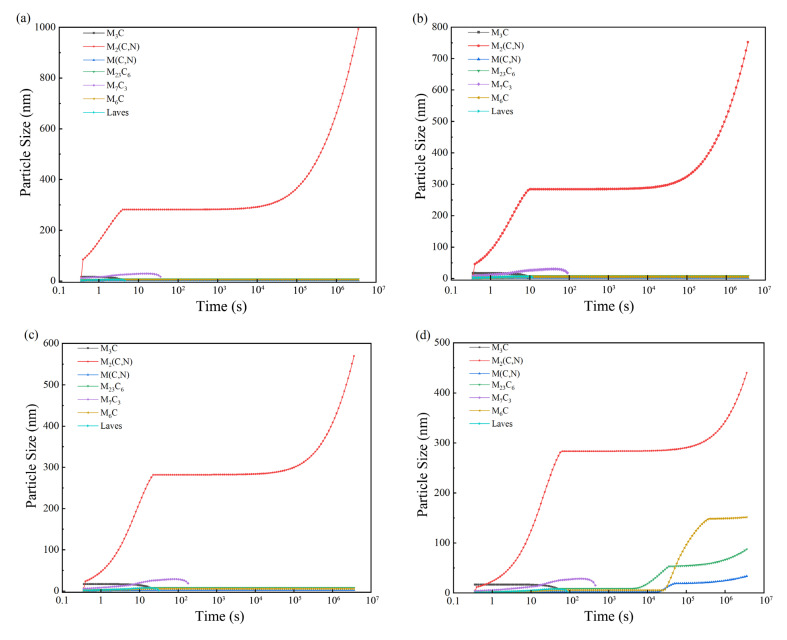

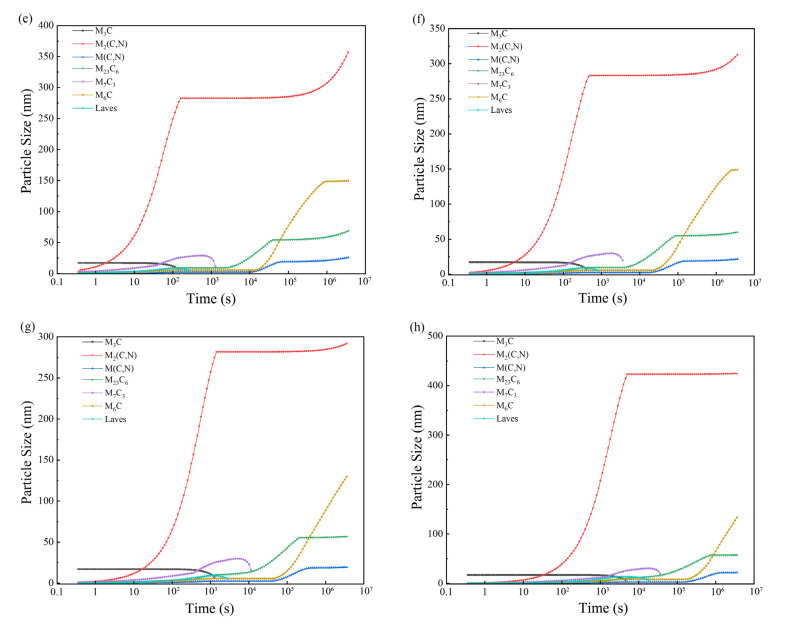
Carbide dimensions of Fe—3.0, Cr—0.3, Mn—7.0, Mo—0.4, Ni—0.3, Si—7.0, and V—2.0 high-speed steel at different tempering temperatures: (**a**) 770 °C; (**b**) 740 °C; (**c**) 710 °C; (**d**) 680 °C; (**e**) 650 °C; (**f**) 620 °C; (**g**) 590 °C; (**h**) 560 °C.

**Figure 6:**
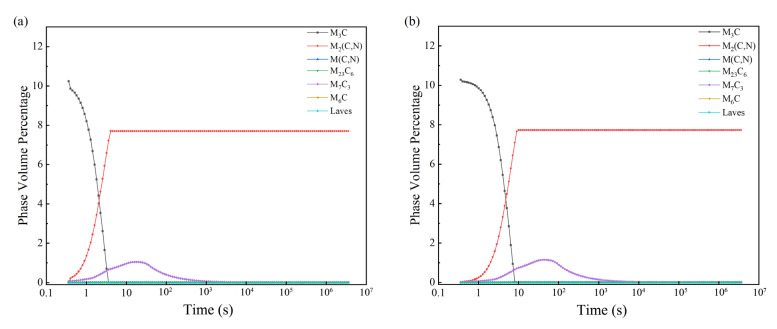

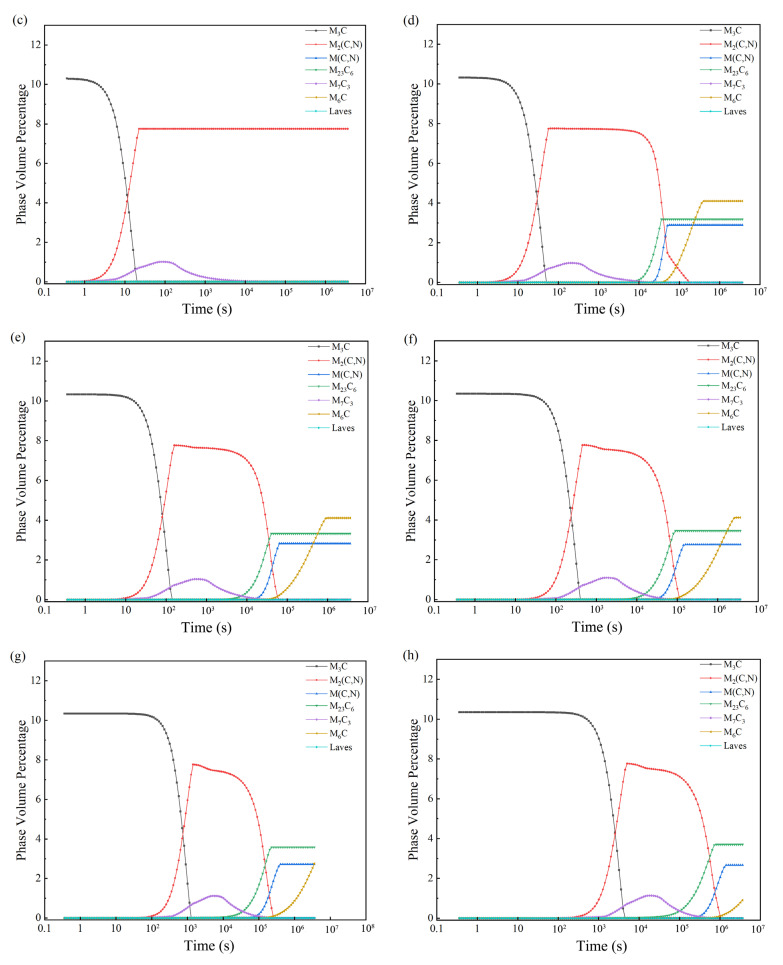
Carbide compositions of Fe—3.0, Cr—0.3, Mn—7.0, Mo—0.4, Ni—0.3, Si—7.0, and V—2.0 for high-speed steel at different tempering temperatures: (**a**) 770 °C; (**b**) 740 °C; (**c**) 710 °C; (**d**) 680 °C; (**e**) 650 °C; (**f**) 620 °C; (**g**) 590 °C; (**h**) 560 °C.

Tempering is an important process after quenching. Its purpose is to eliminate residual stress after quenching and eliminate residual austenite. Tempering at different temperatures can be obtained from different organizations and by adjusting the performance of steel. For high-molybdenum–vanadium high-speed steel to undergo high-temperature tempering, tempering temperatures should have A_c1_ below a certain temperature. If 30 °C is the gradient, the tempering temperatures would be 770 °C, 740 °C, 710 °C, 680 °C, 650 °C, 620 °C, 590 °C, and 560 °C. The quenching temperature should be 1200 °C. Figure 5 and Figure 6 show the results of the precipitation calculations performed in JMatPro.

Figure 5 shows different tempering temperatures for high-molybdenum-vanadium high-speed steel carbide’s particle size changes. Figure 5a–c shows that the tempering temperatures of 710–770 °C have almost no effect on carbide size. Figure 5d–h shows that the size of all carbides increases when the temperature drops to 680 °C, reducing the material’s wear resistance. Figure 6 shows the carbide composition of high-molybdenum-vanadium high-speed steel under different tempering temperatures. Figure 6a–c shows the high-molybdenum-vanadium high-speed steel carbide composition when the tempering temperatures are >710 °C. Reduced temperatures had no significant effect on the material’s carbide content. Figure 6d–h shows that the M_3_C-type content is significantly reduced when the temperature reaches 680 °C, reducing the material’s tempering stability. Other carbide types show an upward trend, increasing the hardness of the material. In summary, high-molybdenum–vanadium high-speed steel tempering temperatures should be set between 550 and 600 °C.

### 4.3. Microstructure Analysis after Heat Treatment Process

**Figure 7:**
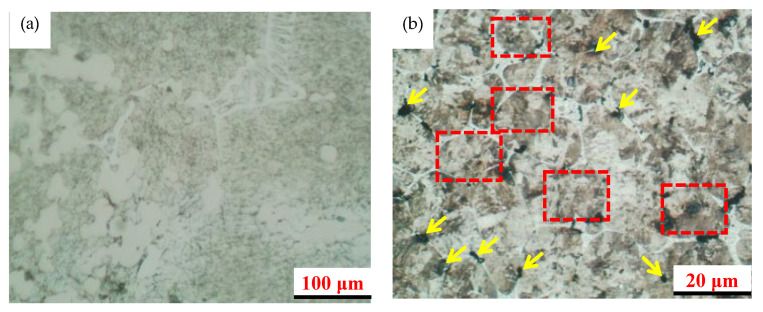
SEM photo of quenching process at 1170 °C: (**a**) ×100, (**b**) ×500 (the red rectangles indicate M_6_C compounds and the yellow arrows indicate VC grains).

**Figure 8:**
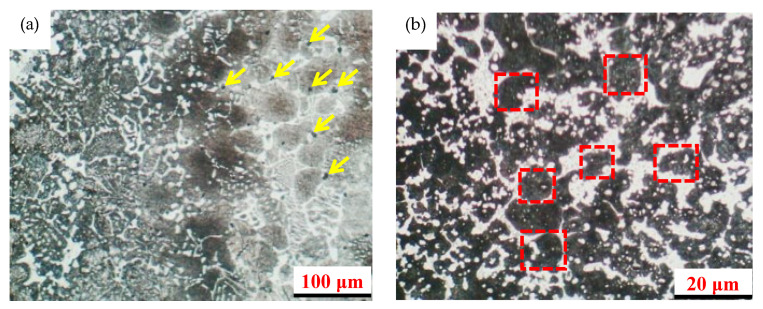
SEM photo of quenching process at 1190 °C: (**a**) ×100, (**b**) ×500 (the red rectangles indicate M6C compounds and the yellow arrows indicate VC grains).

The high-molybdenum-vanadium high-speed steel roll was designed using JMatPro (version 7.0) software for simulation and analysis, the high molybdenum-vanadium high-speed steel meeting the requirements of various components was prepared for smelting, the heat treatment process parameters studied above were applied to the heat treatment at 1170 °C and 1190 °C, respectively, and then the microstructure analysis was carried out. The results are shown in Figure 7 and Figure 8. Figure 7 shows SEM photos of the quenching process at 1170 °C, 560 °C tempering twice, and 570 °C tempering once, respectively. It can be clearly seen from Figure 7 that some mesh-like M_6_C compounds and several small granular VCs are distributed on the matrix of tempered martensite, and the austenite grains can be further refined during the quenching process. After 560 °C tempering twice and 570 °C tempering, most of the residual austenite is converted to tempered martensite, and the properties of the steel are further improved.

Figure 8 shows SEM photos of the quenching process at 1190 °C. Compared with Figure 7 showing heat treatment at 1170 °C, it can be seen that after heat treatment at different temperatures, some carbides in high-speed steel precipitated, the as-cast structure is based on tempered martensite, the M_6_C compound has a finer network structure, and the VC grain distribution is more uniform. It can be concluded that using JMatPro (version 7.0) software to design ingredient formulas has important reference significance for guiding practical production.

## 5. Conclusions

We used JMatPro’s material simulation calculations to calculate high-molybdenum-vanadium high-speed steel roll manufacturing and heat treatment process parameters according to the phase diagram method. The following conclusions were drawn:

(1) High-alloying elements in high-speed steel carbide types with several different carbides or elements produce different performance characteristics in high-speed steel. The design needs to strictly refer to empirical data.
(2) The peak austenite content increases and then decreases as the carbon content increases. However, the carbon content in the austenite increases as the overall carbon content increases. Therefore, the carbon content is determined primarily by its effect on the austenite and martensite transition temperatures.
(3) The difference between the carbon content obtained by software simulation and empirical formulas is because the empirical formula does not consider uncontrollable factors such as humidity and temperature in the actual production process. The error is within the controllable range; however, in the actual production process, actual environmental factors should be considered in addition to theoretical calculations.
(4) Regarding high-molybdenum-vanadium high-speed steel-cast state organization by the martensitic matrix, M(C,N) carbides are more important than residual austenite and various types of carbides. Below 1250 °C, the vanadium carbide content is almost unchanged. Granular vanadium was distributed in a fine dispersion. After tempering, the dispersion treatment was even greater, resulting in secondary hardening that can greatly improve the strength, hardness, and abrasion resistance of high-speed steel.
(5) We simulated high-molybdenum-vanadium high-speed steel-roll casting temperatures of 1360–1410 °C, quenching temperatures of 1190–1200 °C, annealing temperatures of 818–838 °C, and tempering temperatures of 550–600 °C. The heat treatment of the matrix after distributing a large number of small carbides significantly improved wear resistance to meet usage requirements.

## Figures and Tables

**Table 1:** Austenite parameters corresponding to different carbon contents.

Carbon Content (%)	Austenitic Peak (%)	Peak Austenitic Temperature Range (°C)	Austenitic Carbon Content (%)
1.7	91.62	1180–1280	0.38–0.57
1.8	91.15	1170–1270	0.4–0.61
1.9	90.7	1170–1270	0.45–0.65
2.0	90.26	1160–1260	0.48–0.69
2.1	89.84	1160–1260	0.54–0.74
2.2	89.43	1150–1250	0.58–0.79
2.3	89.03	1140–1240	0.63–0.84
2.4	88.66	1140–1240	0.7–0.89
2.5	88.3	1130–1230	0.75–0.95
2.6	87.95	1120–1220	0.81–1.01
2.7	87.62	1110–1210	0.85–1.07

**Table 2:** M50 (°C) temperature at 2.0% carbon content.

Temperature (°C)	Austenite Content (%)	Ms (°C)	M50 (°C)
1160	88.76	191.0	150.7
1170	88.89	183.3	142.7
1180	89.03	175.5	134.6
1190	89.17	167.5	126.2
1200	89.32	159.4	117.8
1210	89.47	151.2	109.1
1220	89.62	142.7	100.3
1230	89.78	134.2	91.2
1240	89.94	125.4	82.0
1250	90.11	116.5	72.6
1260	89.15	110.5	66.2

**Table 3:** M50 (°C) temperature at 2.1% carbon content.

Temperature (°C)	Austenite Content (%)	Ms (°C)	M50 (°C)
1160	88.41	175.5	134.6
1170	88.55	167.6	126.3
1180	88.69	159.6	117.9
1190	88.84	151.4	109.4
1200	88.98	143.1	100.6
1210	89.14	134.6	91.7
1220	89.29	126.0	82.7
1230	89.45	117.3	73.4
1240	89.62	108.3	64.0
1250	89.78	99.2	54.3
1260	83.53	108.22	63.8

**Table 4:** Composition of high-molybdenum–vanadium high-speed steel (wt.%).

C	Mo	V	Cr	W	Mn	Si	Ni	Fe
2.0	7.0	7.0	3.0	0	0.3	0.3	0.4	Bal.

## Data Availability

Data are contained within the article.
